# Differential Effect of Heat Stress on Drought and Salt Tolerance Potential of Quinoa Genotypes: A Physiological and Biochemical Investigation

**DOI:** 10.3390/plants12040774

**Published:** 2023-02-08

**Authors:** Ghulam Abbas, Fiza Areej, Saeed Ahmad Asad, Muhammad Saqib, Muhammad Anwar-ul-Haq, Saira Afzal, Behzad Murtaza, Muhammad Amjad, Muhammad Asif Naeem, Muhammad Akram, Naseem Akhtar, Muhammad Aftab, Kadambot H. M. Siddique

**Affiliations:** 1 Centre for Climate Research and Development (CCRD), COMSATS University Islamabad, Islamabad 45550, Pakistan; 2 Department of Environmental Sciences, COMSATS University Islamabad, Vehari Campus, Vehari 61100, Pakistan; 3 Department of Bio Sciences, COMSATS University Islamabad, Park Road, Islamabad 45550, Pakistan; 4 Institute of Soil and Environmental Sciences, University of Agriculture, Faisalabad 38000, Pakistan; 5 Biochemistry Section, Ayub Agricultural Research Institute, Faisalabad 38000, Pakistan; 6 Soil Chemistry Section, Institute of Soil Chemistry and Environmental Sciences, Ayub Agricultural Research Institute, Faisalabad 38000, Pakistan; 7 The UWA Institute of Agriculture, The University of Western Australia, Perth, WA 6001, Australia

**Keywords:** high temperature, drought, quinoa, salinity, oxidative stress, climate change

## Abstract

Soil salinity, drought, and increasing temperatures are serious environmental issues that drastically reduce crop productivity worldwide. Quinoa (*Chenopodium quinoa* Willd) is an important crop for food security under the changing climate. This study examined the physio-biochemical responses, plant growth, and grain yield of four quinoa genotypes (A7, Titicaca, Vikinga, and Puno) grown in pots containing normal (non-saline) or salt-affected soil exposed to drought and elevated-temperature treatments. Combinations of drought, salinity, and high-temperature stress decreased plant growth and yield more than the individual stresses. The combined drought, salinity, and heat stress treatment decreased the shoot biomass of A7, Puno, Titicaca, and Vikinga by 27, 36, 41, and 50%, respectively, compared to that of control plants. Similar trends were observed for grain yield, chlorophyll contents, and stomatal conductance. The combined application of these three stresses increased Na concentrations but decreased K concentrations in roots and shoots relative to control. Moreover, in the combined salinity, drought, and high-temperature treatment, A7, Puno, Titicaca, and Vikinga had 7.3-, 6.9-, 8-, and 12.6-fold higher hydrogen peroxide contents than control plants. All four quinoa genotypes increased antioxidant enzyme activities (CAT, SOD, and POD) to overcome oxidative stress. Despite A7 producing the highest biomass under stress, it did not translate into increased grain production. We conclude that Puno and Titicaca are more tolerant than Vikinga for cultivation in salt-affected soils prone to drought and heat stress.

## 1. Introduction

More than 20% of irrigated land and 6% of terrestrial areas worldwide are affected by soil salinity, rendering it a serious environmental issue [1]. Pakistan has approximately 10 Mha, or 12.9% of its total land area, that is severely affected by salinity [2], limiting the physiological growth and yield of many field crops [3,4]. During plant growth, soil salinity causes osmotic stress initially, reducing plant water uptake [5,6], followed by nutrient deficiency and ion toxicity. Salinity stress can also decrease gaseous exchange, chlorophyll contents, and root and shoot biomass [7,8]. The extent of salinity damage depends on the salt type, salt concentration, genotype, and plant developmental stage [6,8,9].

According to Shabala et al., (2013) [10], growing salt-tolerant crops could be a profitable use of salt-affected soils. Salt-tolerant plant species have extensive anatomical and morphological traits that strengthen plant performance under saline conditions. Monocotyledonous salt-tolerant plants can tolerate up to 50 mM NaCl, while dicotyledonous halophytes grow successfully at 150 mM NaCl [11,12]. Quinoa is an exemplary dicotyledonous halophyte that can tolerate extremely high levels of salt stress [13,14]. Quinoa genotypes express varying levels of salinity stress tolerance based on their morpho-physiological features [14]. Quinoa genotypes grown worldwide also significantly vary in yield and growth at different latitudes [13,15,16], with unique nutritional characteristics, distinctive amino acid composition, and high protein content [17,18].

Drought is another environmental stressor for plants subjected to severe water stress, especially in semi-arid and arid environments [19]. Water scarcity makes crop production difficult, resulting in significant decline in crop yield [20]. Quinoa has low water requirements and can successfully grow under drought stress [21]. During dry periods, quinoa can maintain leaf area and regulate photosynthetic activity [21,22], using structural features such as small, thin-walled cells, tissue flexibility, low osmotic potential, and dehiscence, which reduces leaf area [22,23]. Other quinoa features for sustaining turgor pressure through osmotic adjustment during drought stress include inorganic ion buildup (e.g., Ca, K, and Na) and improved organic element synthesis (carotenoids and proline) [22,24]. Quinoa genotypes have several drought-resistance mechanisms, enabling this crop to grow and develop under severe drought conditions [25,26].

Climate change has become a significant challenge for agriculture. Along with edaphic factors and other environmental stresses, climate-triggered high temperatures hamper crop productivity. Warmer climatic conditions shorten phenological phases and accelerate plant development, which can lower crop yields [27]. Like that of most crop plants, the pollination stage of quinoa is susceptible to high-temperature stress, which can render the plant infertile [28]. The optimal temperature for quinoa growth is 25 °C, with any upward change in temperature at the flowering stage reducing grain production [28]. Hinojosa et al. (2019a) [29] reported that daytime temperature changes from 25 °C to 40 °C reduced pollen viability by 63%, significantly decreasing seed formation. Increased temperature to 38 °C inhibited seed germination by >50%, with yield losses as high as 30% [28]. High temperatures also significantly decreased quinoa straw yield [30].

Soil salinity, drought, and high temperatures are serious environmental problems in Pakistan. To successfully cultivate quinoa in salt-affected soils prone to drought and high temperature, genotypic variations in quinoa for salt, drought, and high-temperature tolerance need to be assessed. This study aimed to: (1) evaluate the effects of salinity, drought, and high temperature on the growth and yield of various quinoa genotypes; (2) explore physiological and biochemical mechanisms of salinity, drought, and high-temperature tolerance in various quinoa genotypes; (3) recommend the best quinoa genotypes for cultivation in salt-affected soils facing drought and temperature extremes.

## 2. Results

### 2.1. Plant Growth and Biomass

Salinity stress decreased plant growth and the biological yield of the four quinoa genotypes (Table 1). The combined application of salt and drought stress further reduced plant growth and yield, especially with the inclusion of high-temperature stress. Root and shoot lengths of the four genotypes were ranked A7 > Puno > Titicaca > Vikinga under the combination of three stresses. The combined salinity, drought, and high-temperature stress decreased shoot lengths by 28, 30, 36, and 51% and root lengths by 25, 35, 40, and 53% in A7, Puno, Titicaca, and Vikinga, respectively, relative to control plants. A similar pattern was observed for root and shoot biomass.

### 2.2. Grain Yield

Surprisingly, A7 did not produce seeds, while the other three cultivars had viable seed yield. Combined drought and salinity stress decreased seed yield (seed weight and number). Puno had the highest grain weight and seed counts, followed by Titicaca and Vikinga. The combined salinity, drought, and high-temperature treatment had the lowest seed production (Table 2), decreasing seed weights by 35, 45, and 53% and seed numbers by 36, 41, and 51% in Puno, Titicaca, and Vikinga, respectively, compared to control plants.

### 2.3. Chlorophyll Contents

**Figure 1:**
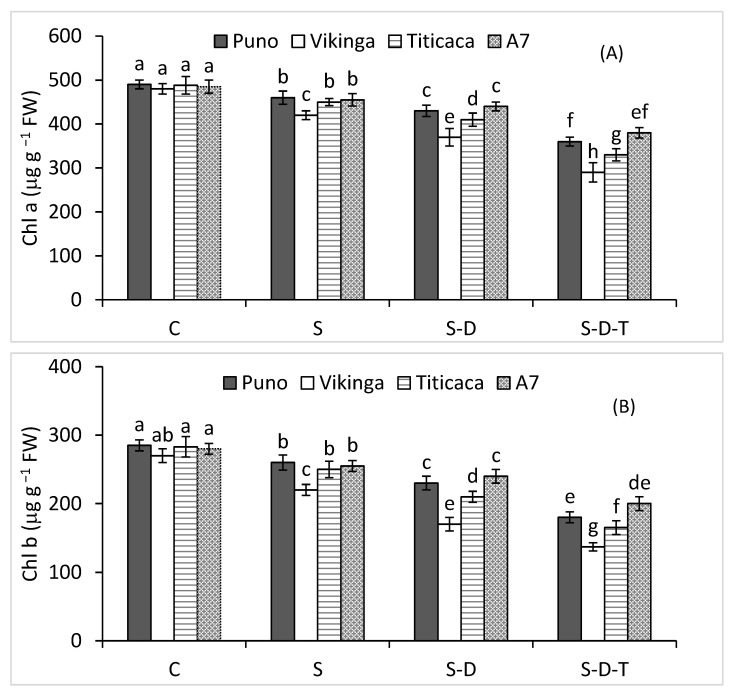

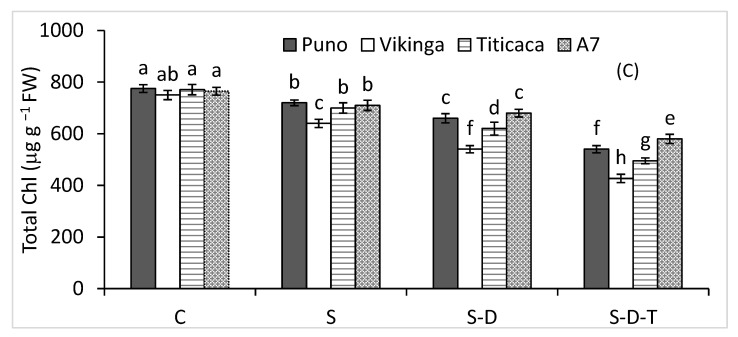
(**A**) Chlorophyll a, (**B**) chlorophyll b, and (**C**) total chlorophyll concentrations of quinoa genotypes under control (C), salinity (S), drought (D), and high-temperature (T) stress. S-D, combined salinity and drought treatment; S-D-T, combined salinity, drought, and high-temperature treatment. The values for each parameter are the means of three replicates ± SE. At the 5% probability level, bars with different letters indicate significant differences.

Chlorophyll contents (total Chl, Chl a, and Chl b) varied significantly across all four quinoa genotypes (Figure 1A–C). A7 and Puno had higher chlorophyll contents than Titicaca and Vikinga under the three stresses applied alone or combined. Salinity alone or combined with drought and high-temperature stress significantly decreased chlorophyll contents in all genotypes. Chl a, Chl b, and total Chl decreased more in Puno (26, 36, and 30%) than in A7 (22, 28, and 24%) under the combined salinity, drought, and high-temperature stress. The corresponding reductions in Titicaca were 32, 42, and 36% and, in Vikinga, were 40, 49, and 43%.

### 2.4. Stomatal Conductance and Relative Water Content

**Figure 2:**
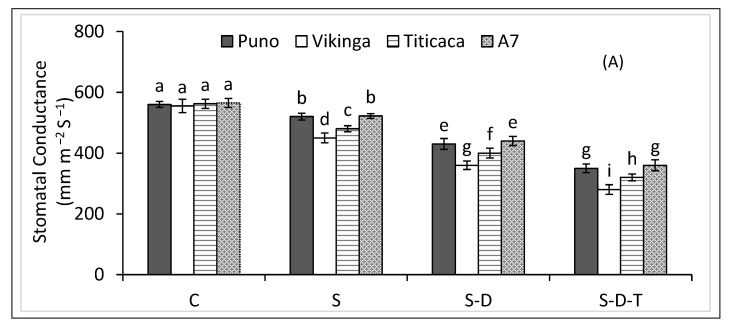

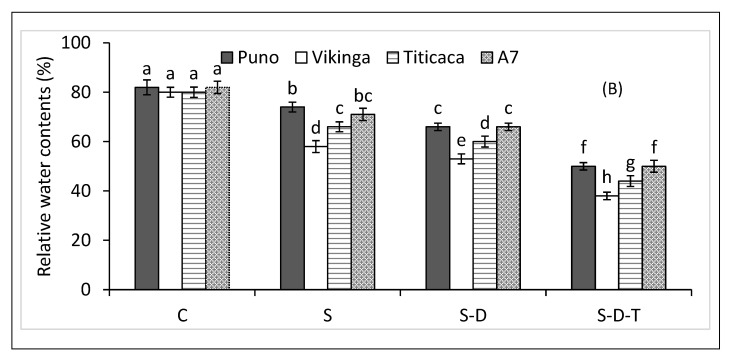
(**A**) Stomatal conductance and (**B**) relative water contents of quinoa genotypes under control (C), salinity (S), drought (D), and high-temperature (T) stress. S-D, combined salinity and drought treatment; S-D-T, combined salinity, drought, and high-temperature treatment. The values for each parameter are the means of three replicates ± SE. At the 5% probability level, bars with different letters indicate significant differences.

Salinity stress reduced stomatal conductance and relative water content in all four quinoa genotypes (Figure 2A,B), more so when combined with drought and/or high-temperature stress. The combined salinity, drought, and high-temperature treatment decreased stomatal conductance by 36, 38, 43, and 50% and relative water content by 39, 39, 45, and 52% in A7, Puno, Titicaca, and Vikinga, respectively, compared to the control.

### 2.5. Shoot and Root Na Concentrations

**Figure 3:**
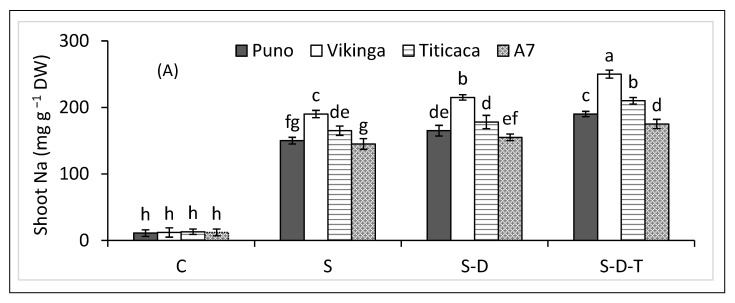

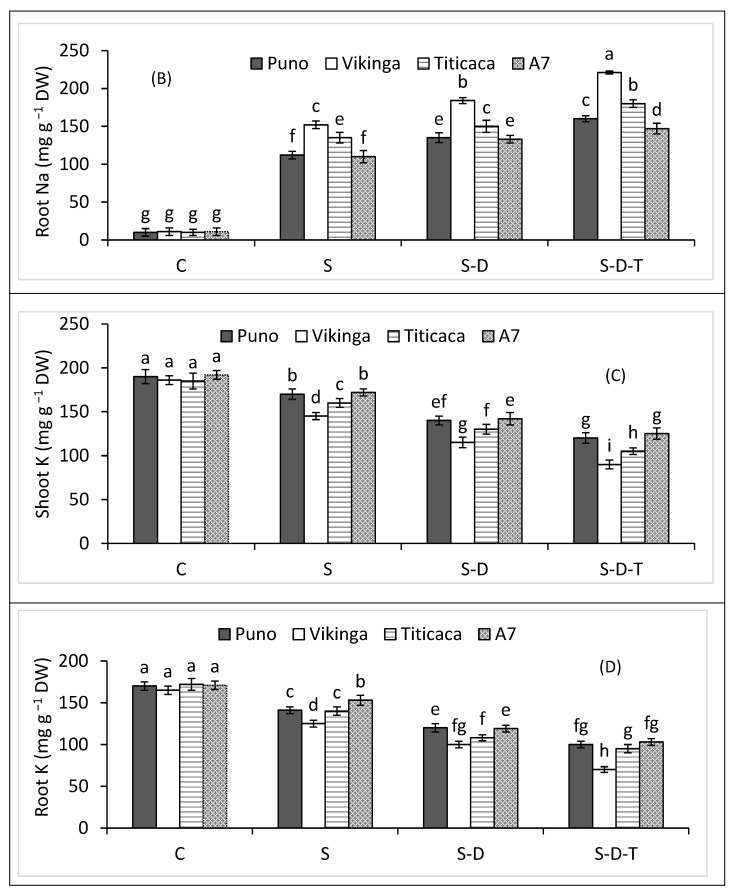
(**A**) Shoot Na, (**B**) root Na, (**C**) shoot K, and (**D**) root K of quinoa genotypes under control (C), salinity (S), drought (D), and high-temperature (T) stress. S-D, combined salinity and drought treatment; S-D-T, combined salinity, drought, and high-temperature treatment. The values for each parameter are the means of three replicates ± SE. At the 5% probability level, bars with different letters indicate significant differences.

Salt stress significantly increased shoot and root Na concentrations in the four quinoa genotypes (Figure 3A,B), more so when combined with drought and/or temperature stress. Vikinga had the highest root and shoot Na concentrations, followed by Titicaca, Puno, and A7 under salinity alone and combined with drought and/or high-temperature stress.

### 2.6. Shoot and Root K Concentrations

Salt stress decreased shoot and root K concentrations, more so when combined with drought and/or temperature stress (Figure 3C,D). A7 had the highest shoot and root K concentrations, while Vikinga had the lowest.

### 2.7. Oxidative Stress

**Figure 4:**
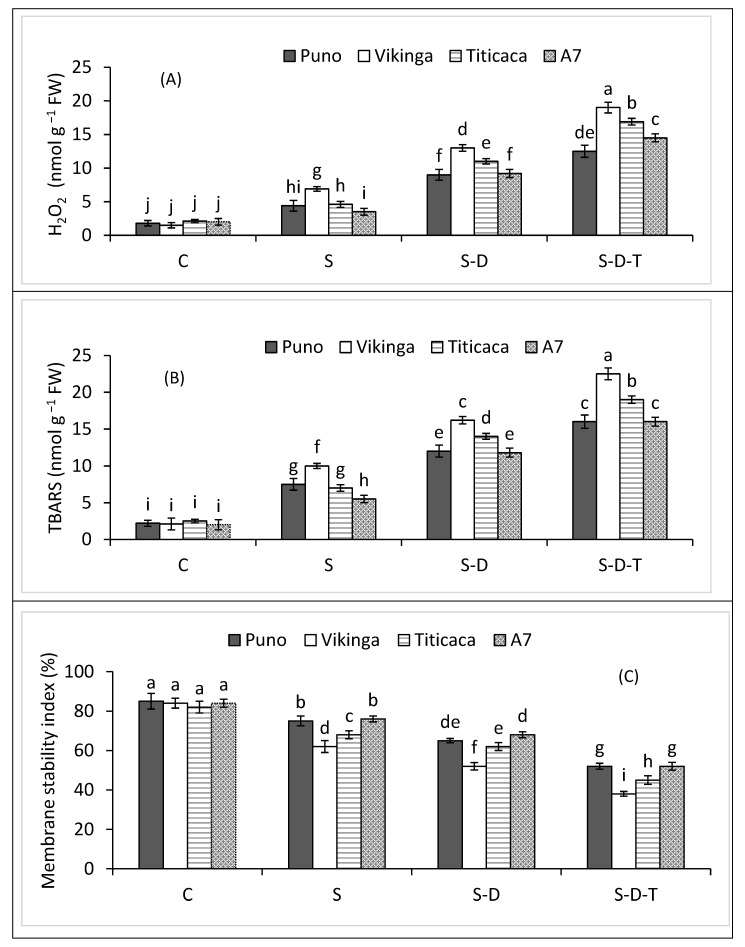
(**A**) H_2_O_2_ contents, (**B**) TBARS contents, and (**C**) membrane stability index of quinoa genotypes under control (C), salinity (S), drought (D), and high-temperature (T) stress. S-D, combined salinity and drought treatment; S-D-T, combined salinity, drought, and high-temperature treatment. The values for each parameter are the means of three replicates ± SE. At the 5% probability level, bars with different letters indicate significant differences.

Salt stress increased hydrogen peroxide (H_2_O_2_) and thiobarbituric acid reactive substances (TBARS) contents in all quinoa genotypes (Figure 4A,B), with the highest values in the combined salinity, drought, and high-temperature treatment. A7, Puno, Titicaca, and Vikinga had 7.3-, 6.9-, 8-, and 12.6-fold higher H_2_O_2_ contents and 8-, 7-, 7.6-, and 11-fold higher TBARS contents, respectively, than the controls. The combined salinity, drought, and high-temperature treatment decreased cell membrane stability (Figure 4C), with 38, 38, 45, and 55% lower membrane stability indexes (MSI) in A7, Puno, Titicaca, and Vikinga, respectively, than in the control.

### 2.8. Antioxidant Enzymes

**Figure 5:**
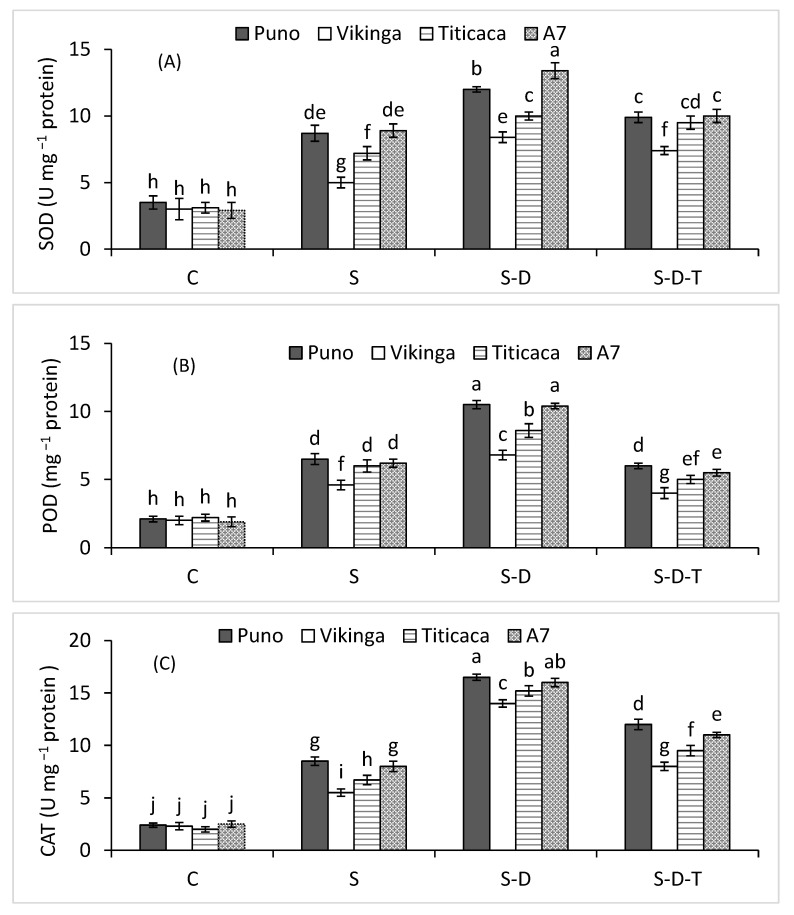
(**A**) SOD, (**B**) POD, and (**C**) CAT activities of quinoa genotypes under control (C), salinity (S), drought (D), and high-temperature (T) stress. S-D, combined salinity and drought treatment; S-D-T, combined salinity, drought, and high-temperature treatment. The values for each parameter are the means of three replicates ± SE. At the 5% probability level, bars with different letters indicate significant differences.

Salt stress boosted superoxide dismutase (SOD), peroxidase (POD), and catalase (CAT) activities (Figure 5A–C), more so when combined with drought stress. In contrast, the combined salinity, drought, and high-temperature treatment decreased SOD, POD, and CAT activities. A7 and Puno had the highest SOD, POD, and CAT activities in the individual salt stress treatment and when combined with drought or high-temperature stress. Vikinga had the lowest enzyme activities in all the treatments.

### 2.9. Multivariate Analyses

**Figure 6:**
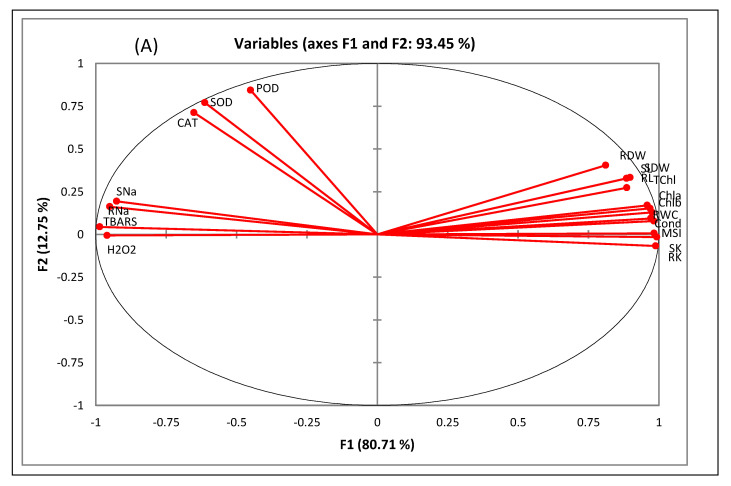

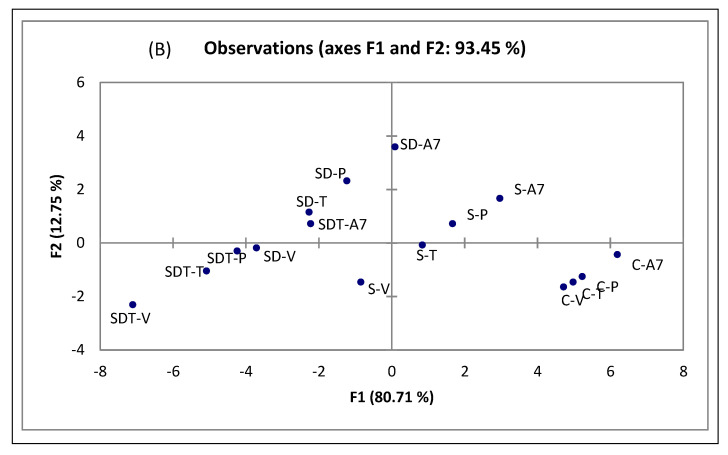
Principal component analysis of (**A**) different response variables and (**B**) different treatments and genotypes of quinoa grown under salinity, drought, and heat stress.

Pearson’s correlation and principal component analysis (PCA) were used to ascertain correlations between response variables and genotypes under salinity, drought, and heat stress (Figure 6, Table 3). The PCA divided the variables into 15 factors (F1 to F15), with four components contributing 81%, 13%, 4.4%, and 1.3% of the total variability. The variables were clustered into three groups: (1) H_2_O_2_, TBARS, and shoot and root Na concentrations; (2) antioxidant enzymes; (3) remaining growth and physiological characteristics (Figure 6A). The genotypes responded differently to the salinity, drought, and heat stresses (Figure 6B). The four genotypes scattered around the positive *x*-axis in the control and salinity treatment and the negative *x*-axis in the combined salinity, drought, and heat stress. Pearson’s correlation matrix showed the correlations between all response variables (Table 3). Plant growth and physiological characteristics negatively correlated with Na contents and oxidative stress attributes.

## 3. Discussion

This study determined the effects of salt, drought, and high temperatures on the growth, grain yield, and physiological characteristics of four quinoa genotypes: A7, Puno, Vikinga, and Titicaca. The four genotypes expressed variable responses when exposed to salinity stress alone or combined with drought and high temperature. The saline–sodic soil severely hampered plant growth and biological yield of all the tested genotypes compared to the normal soil. Genotype A7 performed exceptionally well for biomass production under salinity stress alone and combined with drought and high temperature. In contrast, Vikinga produced the lowest root and aboveground biomass.

Quinoa is a salt-tolerant plant [31], but high salt concentrations and sodicity reduce biomass and grain yield [6,16]. Poor soil physical properties and a higher quantity of exchangeable Na than Ca and Mg in soil exchange sites are the main reasons for the poor growth performance of quinoa in salt-affected soils [6]. Our results demonstrated that salinity combined with drought and high-temperature stress further reduces plant biomass and grain yield. Previous studies have reported the harmful effects of drought stress on quinoa [21,32]. Yang et al. (2020) [33] reported that combined salinity and drought effects were more detrimental to quinoa physiological attributes and plant growth than either stress applied alone. Even though quinoa is a relatively drought-tolerant crop, severe water stress can reduce its biomass and yield [32], the extent of which depends on the drought intensity, genotype, and other environmental factors [32]. Iqbal et al. (2018) reported genotypic differences in quinoa biomass and yield under drought stress, attributing them to oxidative damage due to the overproduction of various ROS. In the present study, A7, Puno, and Titicaca tolerated the combined salinity and drought treatment better than Vikinga, which produced little biomass and grain.

Adverse effects of high temperatures have been reported for various quinoa genotypes [28,29,34]. For instance, high temperatures at anthesis accompanied by drought stress limited grain production in Titicaca [28]. According to Hinojosa et al. (2019a) [29], daytime temperature changes from 25 °C to 40 °C decreased pollen viability by up to 63%, significantly decreasing seed formation. Temperatures above 38 °C can impede quinoa grain yield by up to 50% [28]. Matías et al. (2021a) reported that high temperatures significantly decreased straw yield yet improved the feed value of quinoa due to compositional changes.

Genotypes with better salinity and drought tolerance (A7 and Puno) also performed better under combined salt, drought, and high-temperature stress. Surprisingly, A7 produced considerably higher biomass than the other genotypes but no seeds, possibly due to the long growing season and late maturity, with temperatures above 35 °C during the pollination stage. Vikinga was the most sensitive genotype, producing the lowest biomass and grain yield when subjected to combined salinity, drought, and high-temperature stress.

Physiological attributes, including chlorophyll contents, relative water contents, and stomatal conductance decreased in quinoa under salinity or drought stress, further decreasing under the combined three stresses. Other studies have reported reductions in these attributes in quinoa under salinity [6,35], drought [32,33,36] and high-temperature [21,28,36] stress, which may be due to Na toxicity, water uptake limitations, oxidative stress, or their combinations [32].

When quinoa was grown on saline–sodic soil, root and shoot Na concentrations increased, more so when combined with drought and high-temperature stress, as reported by Iqbal et al. (2018) and Yang et al. (2020). According to Munns and Tester (2008), the toxic effects of Na can be mitigated by decreasing root Na accumulation, reducing Na transport to xylem tissues, reallocating Na from shoots to roots, and sequestering Na into vacuoles. Quinoa genotypes use various methods to deal with excessive Na levels in the rhizosphere [12,37], with some excluding or accumulating excessive Na in their vacuoles. Tolerant genotypes accumulate less shoot and root Na than sensitive genotypes [6]. In the current study, the four quinoa genotypes accumulated more Na in shoots than roots, indicating that excess Na ions were sequestered in leaf vacuoles. Excessive Na taken up by plants can be excluded by Na^+^/H^+^ exchangers present in root cells [31,38,39] or sequestered within vacuoles by NHXNa^+^/H^+^ exchangers present on the tonoplast [31]. The degree of expression of these genes varies among quinoa genotypes [31]. Moreover, Na^+^/H^+^ exchangers are upregulated more in quinoa leaves than roots. Hence, vacuolar Na compartmentation is a more conspicuous mechanism of salt tolerance than root-level exclusion in quinoa [10,39]. In salt-loving plants, inorganic mineral ions (e.g., Na) are used for turgor maintenance of shoot tissues under salt stress [40].

In contrast to Na, tissue K contents decreased when plants were grown in salt-affected soil, more so when combined with drought or drought and high-temperature stress, as reported elsewhere [6,32,33]. Na ions enter cells via K channels during salinity stress [5]. This competitive uptake of Na decreases K contents, leading to the deficiency of this crucial element [3,41,42]. Potassium performs numerous critical physiological functions in plants, including activating more than 50 enzymes [43,44], most of which have a vital role in the biosynthesis of chlorophyll molecules [44]. Additionally, many proteases are over-activated under salinity stress due to K leakage from mesophyll cells, resulting in programmed cell death [45,46,47]. Therefore, the potential of plants to limit K losses and maintain a relatively high K concentration in the cytoplasm directly relates to salt tolerance potential [3,14,29,48]. Our findings corroborate these observations; genotypes with higher K and lower Na in shoot and root tissues (A7, Puno, and Titicaca) produced more biomass than the genotype with higher cellular Na and lower K concentrations (Vikinga).

Salt stress, alone or combined with drought and high-temperature stresses, caused oxidative stress in the four quinoa genotypes in the form of excess H_2_O_2_ production, resulting in lipid peroxidation (MDA) and decreased MSI, as reported elsewhere for other plant species under salinity, drought, and heat stress [29,32,49]. Hinojosa et al. (2019b) [50] also reported that quinoa genotypes increased H_2_O_2_ production under combined drought and high-temperature stress compared to under the individual stresses, and those with lower H_2_O_2_ and oxidative stress produced more biomass and grain yield. We also found that A7, Puno, and Titicaca tolerated oxidative stress more than Vikinga. Various antioxidant non-enzymes and enzymes can reduce ROS-induced oxidative stress [6], including SOD, CAT, and POD [51,52]. ROS detoxification under different environmental stresses occurs in a synchronized manner [6,51]. According to Iftikhar et al. (2022), SOD is critical for detoxifying superoxide (O_2_^•–^) radicals to H_2_O_2_ and O_2_. In all genotypes, salinity alone or combined with drought stress increased SOD activity, more so in A7, Puno, and Titicaca than Vikinga. Salinity, drought, and heat stresses increase SOD activity in various plant species, including quinoa [32,51,53]. H_2_O_2_ is a highly toxic ROS, warranting its detoxification to safeguard cells. CAT and POD help to neutralize H_2_O_2_ by converting it into H_2_O and O_2_ [6]. In the current research, salinity alone or combined with drought increased CAT and POD activities in all four quinoa genotypes, similar to other studies involving stressful conditions [32,51,54,55]. However, high-temperature stress combined with drought and salt stress decreased antioxidant activities, indicating that the combined salt, drought, and high-temperature stress adversely affected the antioxidant defense systems of the studied quinoa genotypes. A7, Puno, and Titicaca had better tolerance against oxidative stress than Vikinga and, thus, performed better under the combined salinity, drought, and high-temperature stress. The sensitivity of Vikinga to these stresses could be attributed to its lower potential to withstand oxidative stress.

Multivariate analyses determined the relationship between genotypes, treatments, and response variables. As previously stated, data analysis using this technique is ideal for depicting covariance and correlations among various variables [6,35]. Shoot and root Na contents negatively correlated with plant growth and physiological attributes, while oxidative stress levels (H_2_O_2_ and TBARS) positively correlated with shoot and root Na concentrations. Antioxidant enzymes positively correlated with oxidative stress characteristics and Na contents. The four genotypes performed differently in different treatments, as demonstrated by their clustering pattern. Genotypes in the control and salt treatments clustered on the positive *x*-axis, while all genotypes with combined salinity, drought, and heat stress clustered on the negative *x*-axis. The results were consistent with the opposite scattering of different treatments with four genotypes. We observed that A7, Puno, and Titicaca performed better in terms of plant growth and physiological attributes than Vikinga, with the combined salinity, drought, and heat stress being more detrimental than the individual stresses.

## 4. Materials and Methods

### 4.1. Plant Material and Treatments

Salt-affected and normal (non-saline) soils were taken from respective fields in the Vehari District of Punjab province, Pakistan, and analyzed for different physicochemical properties (Table 4) before we filled 48 pots with 10 kg soil in each pot.

Recommended rates of N, P, and K fertilizers (120, 90, and 30 kg ha^–1^, respectively) were added to each pot, as per [56] Razzaghi et al. (2012). The Department of Environmental Sciences, CUI Vehari Campus, provided seeds of four quinoa genotypes (Puno, Vikinga, Titicaca, and A7). Each pot was sown with four seeds and thinned to two plants after emergence.

For drought stress investigations, full irrigation (FI) treatments were maintained at 70% soil water-holding capacity, while deficit irrigation (DI) treatments were applied until anthesis before withholding irrigation until harvest. For heat stress treatments, pots with salt-affected soils and DI were placed under optimal temperature (33/13 °C day/night, with 8 h 18 min day length) and humidity (49/73% day/night). Heat-stressed pots were placed in a temperature-controlled glasshouse set 5 °C higher than the ambient temperature. The experiment had a total of 48 pots, 12 each for normal soil + FI (control), salt-affected soil + FI (salinity), salt-affected soil + DI (salinity and drought), and salt-affected soil + DI + high temperature (salinity, drought, and heat), with three replicate pots of each genotype in each treatment.

### 4.2. Plant Physiological Attributes

In clear weather, the stomatal conductance of fully grown leaves was measured using a portable SC-1 leaf porometer (Decagon Devices, Pullman, 142 Washington, DC, USA). The membrane stability and relative water content of the same leaves were measured as per Sairam et al. (2002) [57]. Chlorophyll a, b, and total chlorophyll (a + b) contents were quantified using Lichtenthaler’s method (1987) [58].

### 4.3. Harvesting and Elemental Analysis

Experimental plants were destructively harvested in all treatments at full maturity, followed by seed counts and weight measurements. Root and shoot lengths and dry biomass were recorded. For sodium (Na) and potassium (K) analysis, shoot and root samples were ground and digested in an [59] AOAC (1990) developed diacid mixture of HNO_3_ and HClO_4_. After filtering the digested plant material, a flame photometer (BWB-XP5) was used to measure Na and K contents in the filtrate.

### 4.4. Oxidative Stress Attributes (H_2_O_2_ and TBARS Contents)

The H_2_O_2_ contents were determined in freshly collected quinoa leaves. According to [60] Islam et al. (2008), 0.5 g leaf tissue was crushed and homogenized in trichloroacetic acid before centrifuging at 12,000 rpm for 20 min. Another 0.5 g leaf sample was homogenized at 4 °C in a hydroalcoholic solution (80/20 *v*/*v*) to measure lipid peroxidation. The samples were treated with thiobarbituric acid and butyl hydroxytoluene (BHT), incubated at 95 °C, and then centrifuged at 12,000 rpm for 10 min. Hodges et al.’s (1999) [61] method was used to estimate the concentrations of TBARS indicating lipid peroxidation.

### 4.5. Antioxidant Enzymes

To assess enzymatic activity, 250 mg fresh leaf samples were crushed in 0.1 M phosphate buffer (7.0 pH) and centrifuged at 15,000 rpm for 30 min. The techniques of Aebi (1984) [62], Dhindsa et al. (1981) [63], and Hemeda and Klein (1990) [64] were used to determine CAT, SOD, and POD activities, respectively.

### 4.6. Statistical Analyses

The data were analyzed using a two-way analysis of variance (ANOVA), with genotypes and treatments as factors. The least significant difference test was used to compare treatments and genotypes at the 5% probability level (Steel et al. 1997) [65]. Data are presented as the mean of three replicates ± SE. XLSTAT 2014 was used to perform Pearson’s correlations and PCA.

## 5. Conclusions

This study revealed inconsistent responses of four quinoa genotypes exposed to three environmental stresses (salinity, drought, and high temperature) alone and combined. The combined application of the three stresses was more detrimental for the growth, grain yield, and physiological attributes of the quinoa genotypes than the individual stresses. Genotypes A7, Puno, and Titicaca tolerated the three stresses individually or combined more than Vikinga. Genotype A7 did not produce grain when temperatures neared 40 °C. The better tolerance of A7, Puno, and Titicaca to salinity, drought, and high-temperature stress was attributed to the maintenance of ionic homeostasis and protection against oxidative stress by activation of the antioxidant system. The tolerance potential of these genotypes needs further research in salt-affected field environments susceptible to drought and high temperature.

## Figures and Tables

**Table 1:** Effect of salinity (S), drought (D), and temperature stress (T) on plant growth attributes of quinoa genotypes.

Genotypes	Treatments	Shoot Length (cm)	Root Length (cm)	Shoot Dry Weight (g Plant^–1^)	Root Dry Weight (g Plant^–1^)
Puno	C	43 ± 2 c	20 ± 0.8 c	11 ± 0.5 c	1.1 ± 0.06 d
	S	37± 1 d	17 ± 0.6 d	9.5 ± 0.3 d	1.0 ± 0.06 e
	S-D	34 ± 2 de	15 ± 1 e	8.5 ± 0.2 ef	0.9 ± 0.03 f
	S-D-T	30 ± 2 e	13 ± 1 f	7.0 ± 0.4 gh	0.7 ± 0.04 gh
Vikinga	C	41.2 ± 3 c	19 ± 1 c	10 ± 0.4 cd	1.08 ± 0.08 d
	S	30 ± 2 e	13 ± 0.8 f	8.0 ± 0.2 f	0.7 ± 0.05 gh
	S-D	24 ± 1.5 f	11 ± 0.8 g	7.0 ± 0.4 g	0.6 ± 0.04 h
	S-D-T	20 ± 2 g	9.0 ± 0.5 h	5.0 ± 0.4 i	0.5 ± 0.05 i
Titicaca	C	41 ± 2 c	20 ± 0.8 c	10.5 ± 0.5 c	1.1 ± 0.07 d
	S	36.3 ± 1.6 d	15 ± 1 e	9.0 ± 0.4 de	0.9 ± 0.05 f
	S-D	32 ± 2.1 e	13 ± 0.5 f	8.2 ± 0.3 f	0.78 ± 0.05 g
	S-D-T	26 ± 1 f	12 ± 0.4 g	6.2 ± 0.5 h	0.65 ± 0.05 h
A7	C	53 ± 2.5 a	24 ± 1.3 a	13 ± 0.5 a	1.5 ± 0.1 a
	S	48 ± 1 b	22 ± 1 b	12 ± 0.5 b	1.4 ± 0.04 b
	S-D	44 ± 1.2 c	20 ± 0.8 c	11 ± 0.4 c	1.3 ± 0.08 c
	S-D-T	38 ± 2 d	18 ± 0.9 d	9.5 ± 0.6 d	1.1 ± 0.04 d

Data are means of three replicates ± SE. Values with different letters indicate significant differences at the 5% probability level.

**Table 2:** Effect of salinity (S), drought (D), and temperature stress (T) on grain yield attributes of quinoa genotypes.

Variety	Treatments	Seed Weight (g Plant^–1^)	Seed Number (per Plant)
Puno	C	4.167 ± 0.12 a	2500 ± 50 a
	S	3.54 ± 0.08 c	2200 ± 60 c
	S-D	3.267 ± 0.1 d	1900 ± 50 e
	S-D-T	2.7 ± 0.08 ef	1600 ± 45 g
Vikinga	C	3.8 ± 0.08 b	2300 ± 60 b
	S	2.8 ± 0.1 e	1900 ± 70 e
	S-D	2.4 ± 0.08 f	1500 ± 40 gh
	S-D-T	1.8 ± 0.07 h	1130 ± 40 i
Titicaca	C	4 ± 0.07 a	2450 ± 65 a
	S	3.2 ± 0.09 d	2100 ± 56 d
	S-D	2.8 ± 0.08 e	1700 ± 50 f
	S-D-T	2.2 ± 0.08 g	1450 ± 40 h

Data are means of three replicates ± SE. Values with different letters indicate. Significant differences at the 5% probability level.

**Table 3:** Pearson’s correlation matrix of response variables of quinoa genotypes grown under salinity, drought, and heat stress. Values in bold indicate a significant correlation at alpha = 0.05.

Variables	SL	SDW	RL	RDW	SNa	RNa	SK	RK	Chla	Chlb	TChl	Cond	TBARS	RWC	SOD	CAT	POD	H_2_O_2_
SDW	**0.9873**																	
RL	**0.9861**	**0.9782**																
RDW	**0.9823**	**0.9715**	**0.9814**															
SNa	**−0.7668**	**−0.7637**	**−0.7953**	**−0.6857**														
RNa	**−0.7906**	**−0.7905**	**−0.8158**	**−0.7076**	**0.9961**													
SK	**0.8461**	**0.8630**	**0.8406**	**0.7616**	**−0.8973**	**−0.9285**												
RK	**0.8270**	**0.8444**	**0.8297**	**0.7404**	**−0.9139**	**−0.9411**	**0.9914**											
Chla	**0.8381**	**0.8695**	**0.8176**	**0.7582**	**−0.8371**	**−0.8722**	**0.9674**	**0.9518**										
Chlb	**0.8650**	**0.8825**	**0.8524**	**0.7892**	**−0.8635**	**−0.8966**	**0.9798**	**0.9649**	**0.9888**									
TChl	**0.8521**	**0.8776**	**0.8350**	**0.7738**	**−0.8509**	**−0.8853**	**0.9755**	**0.9602**	**0.9979**	**0.9964**								
Cond	**0.8242**	**0.8500**	**0.8180**	**0.7429**	**−0.8730**	**−0.9068**	**0.9948**	**0.9864**	**0.9741**	**0.9801**	**0.9794**							
TBARS	**−0.8199**	**−0.8399**	**−0.8103**	**−0.7277**	**0.8917**	**0.9216**	**−0.9945**	**−0.9937**	**−0.9665**	**−0.9710**	**−0.9711**	**−0.9924**						
RWC	**0.8284**	**0.8535**	**0.8204**	**0.7513**	**−0.8931**	**−0.9197**	**0.9758**	**0.9643**	**0.9788**	**0.9769**	**0.9807**	**0.9805**	**−0.9688**					
SOD	−0.2921	−0.3113	−0.3260	−0.1888	**0.7069**	**0.6955**	**−0.6162**	**−0.6529**	−0.4625	−0.4896	−0.4756	**−0.5982**	**0.6392**	**−0.5241**				
CAT	−0.3932	−0.3846	−0.4357	−0.3083	**0.6955**	**0.6943**	**−0.6538**	**−0.6851**	−0.4737	**−0.5406**	**−0.5042**	**−0.6289**	**0.6619**	**−0.5242**	**0.9354**			
POD	−0.2109	−0.1967	−0.2754	−0.1403	**0.5980**	**0.5758**	−0.4358	−0.4795	−0.2278	−0.2924	−0.2566	−0.3942	0.4412	−0.3079	**0.9193**	**0.9323**		
H_2_O_2_	**−0.7854**	**−0.8110**	**−0.7639**	**−0.6890**	**0.8302**	**0.8678**	**−0.9818**	**−0.9720**	**−0.9691**	**−0.9635**	**−0.9693**	**−0.9882**	**0.9896**	**−0.9619**	**0.5868**	**0.6059**	0.3602	
MSI	**0.8477**	**0.8689**	**0.8374**	**0.7675**	**−0.8948**	**−0.9234**	**0.9862**	**0.9747**	**0.9818**	**0.9825**	**0.9848**	**0.9888**	**−0.9812**	**0.9948**	**−0.5410**	**−0.5556**	−0.3351	**−0.9738**

**Table 4:** Properties of normal (non-saline) and salt-affected soils used in the experiment.

Soil Types	pH	EC ^1^ (dS m ^−1^)	SAR ^2^ (mmol L^−1^)^1/2^	Organic Matter (%)	Lime Content (%)
Non-saline (0–15)	8.22 ± 0.85	1.56 ± 0.02	3.12 ± 0.45	0.40 ± 0.01	5.4 ± 0.8
Non-saline (15–30)	8.11 ± 0.71	1.54 ± 0.03	3.22 ± 0.55	0.39 ± 0.02	5.3 ± 0.9
Salt-affected soil (0–15)	8.64 ± 0.66	10.9 ± 1.2	25.8 ± 2.5	0.41 ± 0.03	5.48 ± 0.6
Salt-affected soil (15–30)	8.61 ± 0.92	9.5 ± 1.3	22.9 ± 2.85	0.40 ± 0.02	5.44 ± 0.7

^1^ electrical conductivity, ^2^ sodium adsorption ratio of 0–15 cm and 15–30 cm soil depth.

## Data Availability

All data are included in this paper. Additional information can be provided upon request to the correspondence author.
